# SDHI Fungicide Toxicity and Associated Adverse Outcome Pathways: What Can Zebrafish Tell Us?

**DOI:** 10.3390/ijms222212362

**Published:** 2021-11-16

**Authors:** Constantin Yanicostas, Nadia Soussi-Yanicostas

**Affiliations:** Inserm U1141, NeuroDiderot, Université de Paris, F-75019 Paris, France

**Keywords:** succinate dehydrogenase inhibitors, SDHIs, fungicide, zebrafish, bixafen, boscalid, fluxapyroxad, flutolanil, isoflucypram, isopyrazam, penthiopyrad, sedaxane, thifluzamide, neurodevelopment

## Abstract

Succinate dehydrogenase inhibitor (SDHI) fungicides are increasingly used in agriculture to combat molds and fungi, two major threats to both food supply and public health. However, the essential requirement for the succinate dehydrogenase (SDH) complex—the molecular target of SDHIs—in energy metabolism for almost all extant eukaryotes and the lack of species specificity of these fungicides raise concerns about their toxicity toward off-target organisms and, more generally, toward the environment. Herein we review the current knowledge on the toxicity toward zebrafish (*Brachydanio rerio*) of nine commonly used SDHI fungicides: bixafen, boscalid, fluxapyroxad, flutolanil, isoflucypram, isopyrazam, penthiopyrad, sedaxane, and thifluzamide. The results indicate that these SDHIs cause multiple adverse effects in embryos, larvae/juveniles, and/or adults, sometimes at developmentally relevant concentrations. Adverse effects include developmental toxicity, cardiovascular abnormalities, liver and kidney damage, oxidative stress, energy deficits, changes in metabolism, microcephaly, axon growth defects, apoptosis, and transcriptome changes, suggesting that glycometabolism deficit, oxidative stress, and apoptosis are critical in the toxicity of most of these SDHIs. However, other adverse outcome pathways, possibly involving unsuspected molecular targets, are also suggested. Lastly, we note that because of their recent arrival on the market, the number of studies addressing the toxicity of these compounds is still scant, emphasizing the need to further investigate the toxicity of all SDHIs currently used and to identify their adverse effects and associated modes of action, both alone and in combination with other pesticides.

## 1. Introduction

Molds and fungi have been a major threat to crops throughout human history. A potato crop fungus caused the Irish potato famine of the 1850s, and mildew mold wrought the complete destruction of French vineyards at the end of the 19th century. Besides crop destruction, fungi also produce mycotoxins, which can contaminate agricultural products and make them unfit for consumption or even toxic, as exemplified by the mass food poisoning in Kenya in 2004 due to the consumption of moldy maize contaminated with aflatoxin, which caused 125 deaths [1]. From the 1950s onward, the chemical industry has developed fungicides to respond to the threats posed by these pests. These agents have since become increasingly widely used in modern agricultural practices worldwide. Today, among the different classes of fungicides, SDHIs are the fastest growing family as seen by the number of new products arriving on the market over the past 10 years [2]. Their fungicidal properties rely on their ability to inhibit the SDH/electron transport chain (ETC) complex II (CII), an essential and evolutionarily conserved mitochondrial enzymatic complex critically required for proper functioning of both the ETC and tricarboxylic acid (TCA)/Krebs cycle, both essential for cellular energy production and ATP synthesis [3].

Importantly, the SDH/CII complex is crucial for energy production not only in fungi, but also in all organisms that have mitochondria (i.e., almost all extant eukaryotes). It was recently shown that eight SDHI fungicides currently used for agricultural purposes are highly efficient inhibitors of SDH activity in off-target species, such as bees, earthworms, and humans [4]. These findings raise concerns about the possible toxicity of these compounds toward non-target organisms and, more generally, toward the environment. According to the Pesticide Properties DataBase of the International Union of Pure and Applied Chemistry (IUPAC), SDHI fungicides display low acute toxicity toward mammals and birds, with acute oral LD_50_ values ranging from 2000 mg/kg to over 6500 mg/kg in rodents and about 2500 mg/kg in birds. SDHIs can be highly toxic to fish, with 96 h LC_50_ values for the adult common carp (*Cyprinus carpio*) as low as 8.7 nM, 70 nM, and 170 nM for benzovindiflupyr, isopyrazam, and isoflucypram, respectively. According to manufacturers’ recommendations, the concentrations of SDHI solutions spread on fields should range from 0.5 to 2 mM, and the initial concentration of thifluzamide in paddy water after spraying is 7.4 µM [4]. In addition, the increasing use of SDHI fungicides worldwide results in these substances being frequently detected in aquatic environments [5], sometimes at concentrations exceeding reported toxic levels (e.g., 0.72 µM flutolanil in effluent water in Japan [6] and 0.1 µM boscalid in coastal estuary water in California [7]).

Over the last two decades, the zebrafish, a small, easily bred freshwater fish, has become increasingly used as a model in many fields of biology, including toxicology. The reasons for the popularity of this vertebrate model are numerous and well-known. They have already been described in many reviews [8] and so will not be discussed here. Suffice to say that the zebrafish has been recommended by the OECD as a model organism to study the toxicity of environment-contaminating chemicals and pesticides [9].

Herein we review all the toxicological investigations carried out so far with the zebrafish on nine SDHI fungicides currently used in agriculture: bixafen, boscalid, fluxapyroxad, flutolanil, isoflucypram, isopyrazam, penthiopyrad, sedaxane, and thifluzamide. Importantly, while the data reveal that many of these compounds induce various adverse effects at environmentally relevant concentrations, this review also points out that there are a great number of new SDHI fungicides in addition to the compounds reviewed here, and that there is a lack of extensive toxicological work on 14 currently used SDHI fungicides, namely, benodanil, benzovindiflupyr, fenfuram, fluindapyr, fluopyram, furametpyr, inpyrfluxam, isofetamid, isoflucypram, mepronil, oxycarboxin, penflufen, pydiflumetofen, and pyraziflumid.

## 2. The SDHI Fungicides—A Fast-Growing Pesticide Family Who’s Toxicity Has Been Scantly Studied

The fungicidal property of SDHIs relies on their ability to inhibit the SDH/CII complex in molds and fungi. This complex is a universal key component of the mitochondrial respiratory chain, which transfers electrons generated during the oxidation of succinate to fumarate to a pool of ubiquinone, which is then reduced to ubiquinol [10]. The SDH/CII complex is therefore crucial for the proper functioning of both the mitochondrion ETC and TCA cycles—two metabolic pathways that are essential for energy supply, cell metabolism, and many other vital processes. Hence, even partial inhibition of SDH activity is expected to cause marked changes in metabolism and have severe adverse consequences for the cells [11]. Importantly, besides the essential and evolutionarily conserved requirement for the SDH/CII complex, the four proteins constituting the enzymatic complex (SDHA to D) and, especially, the quinone binding pocket of the tetrameric complex, which is the molecular target of all carboxin-derived SDHIs, display a high level of evolutionary conservation at both the structural and amino acid sequence levels [4]. As a likely consequence of this conservation of SDH proteins throughout evolution, it has also been shown [3] that eight SDHI fungicides currently used in agriculture are efficient inhibitors of the SDH activity in several off-target species, including bees, earthworms, and humans, with IC_50_ values toward human SDH as low as 0.34 and 0.63 µM in the case of SDHIs bixafen and isopyrazam, respectively.

The first SDHI fungicide, carboxin, was introduced in 1969 to combat basidiomycete fungi, such as rusts and smuts (IUPAC, Pesticide Properties DataBase). However, Mowery et al. [12] investigated the effect of this substance on the activity of SDH extracted from beef heart and demonstrated that carboxin efficiently inhibited this SDH enzymatic activity, with IC_50_ values in the micromolar range. Alongside carboxin and flutolanil, which were first marketed over 30 years ago, a new generation of SDHI fungicides has appeared in the last ten years comprising boscalid, benzovindiflupyr, isopyrazam, penthiopyrad, sedaxane, fluopyram, and others. In 2021, national and international regulatory authorities approved 22 SDHI fungicides worldwide, and 2 are pending authorization (FRAC, 2021, classification of fungicides). In addition, the emergence of resistance to existing SDHIs makes the discovery of fungicides with novel modes of action an urgent need, which should also lead to an increase in the number of new SDHIs in the near future [12]. Of particular importance here, some new-generation SDHI fungicides display biocidal activities that go beyond mold destruction, as illustrated by fluopyram, which is also used as a highly effective nematicide to combat parasitic nematodes in soils and lawns [13]. It is of note that fluopyram caused an increased incidence of thyroid follicular cell adenomas in male mice at 105 mg/kg/day in a mouse oncogenicity study [14]. However, the toxicity of fluopyram toward zebrafish embryos, larvae, or adults has not yet been evaluated.

## 3. Acute Toxicity of SDHI Fungicides

According to the IUPAC Pesticide Properties DataBase, most SDHI fungicides are considered as moderately toxic to fish species; the rainbow trout (*Oncorhynchus mykiss*), the fathead minnow (*Pimephales promelas*), and the common carp have 96 h LC_50_ values of >1 µM. However, four SDHIs display high toxicity to adult fish, namely, benzovindiflupyr, isopyrazam, isoflucypram, and bixafen (96 h LC_50_ 8.7 nM, 70 nM, 170 nM, and 230 nM, respectively) (Table 1 and Table 2).

So far, the 96 h LC_50_ values for freshly fertilized zebrafish embryos (2–6 hpf) have been determined for the SDHIs bixafen [15,16], boscalid [17], flutolanil [18], fluxapyroxad [19], isopyrazam [20], penthiopyrad [21], sedaxane [22], and thifluzamide [23]. The results confirm that most of these compounds display moderate toxicity, with 96 h LC_50_ values ranging from 2 to 17 µM, as shown in Table 1. The one exception is isopyrazam, which was highly toxic toward zebrafish embryos (96 h LC_50_ 140 nM) (20), recalling the high toxicity identified in adult rainbow trout (96 h LC_50_ 70 nM). As shown in Table 1, most acute toxicity data obtained with zebrafish embryos were in close agreement with those reported in the IUPAC database for other fish species. However, differences were also observed. Bixafen was highly toxic to adult rainbow trout (96 h LC_50_ 230 nM) but was only moderately toxic to zebrafish embryos (96 h LC_50_ 2.12 μM (15) and 2.7 µM (16)) (Table 1 and Table 2). However, further work is needed to determine whether these differences are due to species-specific or stage-specific toxicities of the SDHI bixafen.

Because the sensitivity to toxicants may vary according to the stage of individuals, the 96 h LC_50_ values of thifluzamide [23], flutolanil [18], and fluxapyroxad [19] were also determined for zebrafish adults and larvae, and Qian et al. [21] also studied the acute toxicity of penthiopyrad in zebrafish larvae (Table 1).

Analysis of the 96 h LC_50_ values indicated that the most sensitive stage varied according to the SDHI studied. For instance, whereas zebrafish embryos displayed the highest sensitivity to thifluzamide (96 h LC_50_ 5.83 µM) and adults the lowest (96 h LC_50_ 7.93 µM), adults showed the highest sensitivity to flutolanil (96 h LC_50_ 8.35 µM) and embryos the lowest (96 h LC_50_ 16.91 µM), with larvae displaying an intermediate sensitivity in both cases (96 h LC_50_ 6.66 and 12.65 µM, respectively). In the case of fluxapyroxad, larvae showed the lowest sensitivity (96 h LC_50_ 1.83 µM) and embryos the highest (96 h LC_50_ 3.64 µM), with adults displaying an intermediate value (96 h LC_50_ 2.4 µM). Lastly, larvae were found to be slightly more sensitive to penthiopyrad (96 h LC_50_ 6.62 µM) than embryos, (96 h LC_50_ 7.7 µM). Interestingly, in the case of strobilurin fungicides, which target the mitochondrion ETC CIII [24], data indicate that zebrafish larvae and juveniles are globally more sensitive to these compounds than adults and embryos, likely owing to the protective effects of the chorion surrounding embryos and the fully efficient antioxidant system in adults [25]. The differences in the stage-specific toxicity of the SDHIs described above could therefore also reflect specific off-target effects differentially affecting embryos, larvae, or adults. Further work will be needed to fully characterize the toxicity of these compounds toward zebrafish embryos, larvae, and adults, and their associated adverse outcome pathways.

The acute toxicity to adult zebrafish of thifluzamide, flutolanil, and fluxapyroxad also enabled a comparison of the LC_50_ values with those observed for other adult fish species. The results show that the most sensitive species varies according to the SDHI studied. For example, bluegill (*Lepomis macrochirus*) was more sensitive to thifluzamide (96 h LC_50_ 2.46 µM) than zebrafish (96 h LC_50_ 7.93 µM), but less sensitive to flutolanil (96 h LC_50_ 16.7 µM) than zebrafish (96 h LC_50_ 8.35 µM). Another example was fluxapyroxad, for which the 96 h LC_50_ value was 0.76 µM for common carp and 3.02 µM for bluegill. By contrast, the 96 h LC_50_ values of fluxapyroxad were close for adult zebrafish and bluegill (2.4 µM, and 3.02 µM, respectively).

As stated above, nine SDHI fungicides have so far undergone toxicological investigations with zebrafish, and 96 h LC_50_ values were determined for only eight of them, emphasizing the urgent need to further investigate the toxicity of all the currently used SDHIs. For example, benzovindiflupyr, a second-generation SDHI that is highly toxic to fish according to the IUPAC (96 h LC_50_ 8.7 nM for adult common carp), has not yet undergone toxicology investigations with the zebrafish (Table 2). Lastly, the findings reviewed here also demonstrate the suitability of the zebrafish as a model to investigate the toxicity of environmental pollutants towards fish.

## 4. Developmental Toxicity of SDHI Fungicides

Besides the acute toxicity of SDHIs and their LC_50_ values for embryos, larvae, or adults, we know that environmental toxicants can also induce adverse effects impairing various developmental processes. Zebrafish embryos have provided versatile tools to characterize the developmental toxicities of the nine SDHIs reviewed here and also help in deciphering their associated adverse outcome pathways. In particular, because the mode of action of SDHIs is inhibition of SDH/CII in fungi, zebrafish embryos have been instrumental in investigating the effects of these fungicides on mitochondrion metabolism, fatty acid synthesis, and reactive oxygen species (ROS) accumulation. The adverse effects of these SDHIs on the development and functioning of the CNS, and on behavior, are reviewed below.

Embryos exposed to bixafen at 0.9 µM for 48 h showed decreased hatching rate and developmental abnormalities, including tail shortening, spinal curvature, and pericardiac edema [15,16]. In addition, exposure to bixafen at 0.3 µM and above caused markedly decreased pigmentation of the trunk and retina [15,16].

Qian et al. [17] showed that exposure of embryos for 96 h to boscalid at 4.93 µM and above caused severe morphological defects, including yolk sac edema, pericardiac edema, and spine curvature. Exposure to 1.75 and 3.49 µM inhibited swim bladder inflation and induced apoptosis as shown by an increased number of apoptotic cells (1.75 and 3.49 µM) and upregulation of pro-apoptotic genes: *puma* and *apaf-1* (1.75 and 3.49 µM) and *p53*, *bax*, *casp-3*, and *casp-9* (3.49 µM). Boscalid exposure also increased expression of genes related to melanin metabolism (3.49 µM), impaired melanin transport and deposition (0.87 µM), and adversely affected lipid metabolism, with embryos showing increased accumulation of both triglycerides and cholesterol (1.75 µM) and decreased expression of genes involved in lipid metabolism: *ppar*α*1* and *fas* (0.87 µM), *cyp51* (1.75 µM), and *hmgcra* and *acca1* (3.49 µM). Wang et al. [25] used concentrations (14.56, 43.68, and 72.8 µM) well above the 96 h LC_50_ value (7.72 µM) and a 48 h exposure protocol to investigate the adverse effects and modes of action of boscalid. The results first confirmed that boscalid at 14.56 µM and above impaired melanin deposition and, more importantly, increased ATPase and catalase (CAT) activities and reactive oxygen species (ROS) production, combined with a marked decrease in superoxide dismutase (SOD) activity, suggesting that this SDHI caused massive oxidative stress in embryos. Interestingly, the authors also showed that treatment with fullerene, an inhibitor of oxidative stress, at 100, 200, and 400 nM decreased both ROS production and the rate of malformations induced by exposure to 72.8 µM boscalid.

Following exposure to flutolanil at 6.18 µM, Teng et al. [5] observed that 72 hpf embryos failed to hatch and showed severe morphological defects, including pericardial edema, spine curvature, and shortened tail. In addition, flutolanil at 1.57 µM induced slower heartbeat, increased accumulation of thyroid hormones T3 and T4, and expression changes of several genes involved in thyroid hormone transport and regulation: *trh*, *tsrh*, *tpo*, *dio1*, and *tra*. The increased expression of thyroid-stimulating hormone receptor (TSHR) in embryos exposed to flutolanil at 1.57 µM and above was confirmed by Western blotting. Flutolanil at 3.14 µM also induced alterations in energy metabolism (decrease in succinate and maltose), amino acids (reduced levels of tryptophan, histidine, and phenylalanine), and nucleotide synthesis (reduced uracil concentration). Flutolanil exposure thus caused both alterations of various metabolites and thyroid hormone disruption in zebrafish. Yang et al. [18] further explored the effects of flutolanil exposure on the development and circadian cycle of embryos.

Li et al. [26] investigated the adverse effects induced by low doses of fluxapyroxad on zebrafish embryos. The results showed that exposure to a low dose of this SDHI (1 µM) for 48 h caused an increased rate of malformations and a reduced hatching rate. A higher concentration of fluxapyroxad (4 µM) induced significantly decreased pigmentation and blood clotting clustering in 48 hpf embryos. Fluxapyroxad at 1 µM also significantly increased the expression of the ectoderm marker gene *foxb1a*, while it decreased that of the neuronal marker genes *crx* and *NeuroD*. Higher concentrations decreased the expression levels of the *gh* (2 µM) and *nkx2.4b* genes (4 µM). Lastly, a non-significant increase in SOD and CAT activities and malonaldehyde (MDA) content in all treatment groups and an increase in glutathione (GSH) in embryos exposed to 2 and 4 µM fluxapyroxad were observed, suggesting that this SDHI induced oxidative stress. Further work is needed to characterize the effect of fluxapyroxad on oxidative stress.

Chen et al. [27] showed that exposure to isoflucypram at 2.5 µM for 96 h did not increase embryonic lethality but induced severe abnormalities, including yolk sac edema, pericardial edema, blood clotting clustering, hatching delay, and decreased heart rates in zebrafish. In the same study, the authors showed that expression of the *alas2* gene encoding an enzyme essential for hemoglobin synthesis was markedly inhibited following exposure to isoflucypram at 25 nM and above.

Yao et al. [20] investigated the consequences of 96 h exposure of embryos to low doses of isopyrazam. Developmental abnormalities, including edema, small head deformity, body deformation, and decreased pigmentation, were observed in embryos exposed to isopyrazam at 0.07 µM. Moreover, isopyrazam at 0.28 µM and above caused oxidative stress and significantly decreased SDH activity, suggesting that the adverse mode of action of this SDHI includes ETC/TCA inhibition and subsequent oxidative stress.

Qian et al. [21] studied the subacute toxicity of penthiopyrad toward embryos following 96 h of exposure. The results indicated that penthiopyrad at 3.32 µM impaired lipid metabolism, including fatty acid synthesis and ß-oxidation. This SDHI caused pericardiac edema (0.83 µM) and decreased heartbeat and yolk sac edema (1.66 µM). Penthiopyrad also induced downregulation of genes involved in lipid metabolism: *srebf1* (0.83 µM) and *hmgcra*, *ppar*α*1*, *cyp51*, and *acca1* (1.66 µM).

Following exposure for 120 h to sedaxane at 3.17 µM, Yao et al. [22,28] observed increased accumulation of ROS and MDA and increased peroxidase, SOD, and SDH activities, associated with decreased glutathione levels, indicating that embryos experienced oxidative stress. In good agreement with this, the expression of genes associated with oxidative stress—*cat*, *gpx1a*, *sod1*, and *sod2*—was markedly upregulated following exposure to 6.34 µM sedaxane. By contrast, the *sdhb* gene was markedly downregulated following exposure to 3.17 µM sedaxane.

Yang et al. [29] first showed that exposure to a sublethal dose of thifluzamide caused several adverse effects on embryonic development. Embryos exposed to 0.36 µM thifluzamide displayed yolk sac edema and cells showing apoptotic features. The observed effects also included increased interleukin Il-8 content, decreased expression of the gene *sdha*, and upregulation of *polg1*, *tk2*, and *tfam*, three genes involved in mtDNA replication and transcription. Following exposure to thifluzamide at 3.6 and 5.39 µM, severe pathological changes were observed in embryos, including yolk sac and pericardiac edema, uninflated swim bladder, and severe mitochondrion damage. Other defects were detected, such as inhibition of SDH activity, decreased SOD and CAT content, and marked downregulation of genes involved in mtDNA replication, defenses against oxidative stress, and ETC functioning, associated with increased expression of genes related to apoptosis and inflammation. This suggests that adverse effects of thifluzamide on mitochondrion structure and functioning, including inhibition of SDH activity, might be responsible for oxidative damage, inflammation, and, ultimately, cell apoptosis and death.

## 5. Long-Term Toxicity of SDHI Fungicides

The determination of the acute toxicity concentrations inducing adverse effects and the associated modes of action is essential for estimating the dangerousness of pesticides and setting appropriate regulations. However, the characterization of the adverse effects induced following long-term exposure to low doses of any pesticide is much more relevant to the situations encountered in natural environments, and adult zebrafish have been used as tool to evaluate the adverse effects caused by long-term (14–60 days) exposure to low doses of the SDHIs boscalid, flutolanil, and thifluzamide.

The long-term toxicity of boscalid to adult zebrafish was first studied by Qian et al. [30]. The results showed that exposure to boscalid at 0.29 µM for 28 days caused a decrease in weight and length, blood glucose content, hexokinase and SDH activities, and triglyceride content, and an increase in glycogen content in the liver. In individuals exposed to 0.029 µM, a decrease in the activity of fatty acid synthase (FAS) and acetyl coenzyme A carboxylase (ACC), combined with increased expression of the gene encoding G6Pase, was also observed. Lastly, gene expression analysis also confirmed that boscalid at 0.29 µM induced downregulation of *fas* and other genes involved in lipid metabolism, such as *srebp1*, *mgst1*, and *hmgcra*. More recently, Qian et al. [31] observed that the diameters of the adult eye and cornea, together with the photoreceptor layer, were significantly decreased following 21-day exposure to boscalid at 0.29 µM and above.

Teng et al. [32] investigated the chronic toxicity of flutolanil following a 60-day exposure protocol, with a focus on liver physiology and metabolism. First, hepatotoxicity, characterized by infiltrated lymphocytes and hepatocyte vacuolization, was observed in the liver of individuals exposed to 0.77 nM and 0.15 µM flutolanil, respectively. Exposure to flutolanil at 0.77 nM also caused a decrease in CAT activity and an increase in MDA content in both sexes, associated with increased casp-3 accumulation in females. Importantly, both males and females exposed to flutolanil 0.15 µM showed an increase in the content of 8-hydroxy-2-guanosine, 8-OHdG, a marker of genotoxicity and DNA damage. Lastly, upregulation of the pro-apoptotic genes *bax* and *apaf1* was observed in males exposed to 0.77 nM flutolanil, while the expression of these genes, and also of *bcl-2*, was markedly decreased in females exposed to 0.77 nM flutolanil and above. Given that endocrine disruption is a well-described effect of many environmental pollutants, Teng et al. [33] used the same 60-day exposure protocol to investigate the consequences of chronic exposure to flutolanil on endocrine metabolism, hormone synthesis, and gonad development in adults of both sexes. First, consistent with the hepatotoxicity previously observed, decreased liver weight was observed in males and females exposed to 0.15 µM and 3.09 µM flutolanil, respectively. Importantly, the data showed that while testosterone levels were decreased dose-dependently in both males and females exposed to 0.77 nM flutolanil and above, a dose-dependent increase in estradiol concentrations was also observed in these individuals. Gene expression analysis revealed major changes in both males and females exposed to flutolanil at 0.77 nM and above. In females, many genes involved in hormone synthesis and endocrine metabolism (*fshr*, *lhr*, *cyp11a*, *11ßhsd*, *17ßhsd*, *cyp19b*, and *star*) were downregulated in individuals exposed to flutolanil at 0.77 nM and above. In males, the expression of genes involved in steroidogenesis and steroid metabolism was globally upregulated following exposure to flutolanil. Lastly, the data showed that the fecundity of females and the fertility of males were both significantly decreased following exposure to flutolanil at 0.15 and 3.09 µM.

The SDHI that has been most thoroughly studied for long-term toxicity in adult zebrafish is thifluzamide. Yang et al. [23] first showed that following exposure to thifluzamide at 0.36 µM for 21 days, liver damage could be observed, including hepatocyte vacuolization and necrosis. In addition, decreased mitochondrial enzymatic activities were detected in adults exposed for 28 days to thifluzamide at 0.36 µM, including SDH and all four ETC complexes, and exposure to 0.036 µM significantly affected mitochondrion morphology and inhibited ETC complexes III and IV. Lastly, gene expression analysis indicated that thifluzamide at 0.036 µM and above caused decreased expression of immunity-related genes *il-8* and *ifn* and two chemokine-encoding genes. Thifluzamide at 0.036 µM also induced upregulation of pro-apoptotic genes *p53*, *casp-3*, and *apaf1*, but higher doses (0.36 µM and above) induced markedly decreased expression of these genes and of *bcl-2* and *casp-9*. This study produced the first evidence that long-term exposure to low doses of thifluzamide causes severe adverse effects related to mitochondrion defects and subsequent apoptosis. Using the same 28-day exposure protocol, Yang et al. [34,35] further investigated the long-term adverse effects of thifluzamide with a focus on liver toxicity, glycometabolism, energy production, and lipid metabolism. Thifluzamide at 3.6 µM caused a significant increase in liver glycogen content and glucose-6-phosphate dehydrogenase (G6PDH) activity, associated with a marked decrease in blood glucose concentration and lactate dehydrogenase (LDH) activity. Pyruvate accumulation was also significantly decreased following exposure to thifluzamide at 0.36 µM. Gene expression analysis indicated that genes required for mtDNA replication and transcription, *polg1*, *twk*, *tk2*, *polmt*, *tfam*, and *mt-nd1*, were downregulated, and *sdha* was upregulated, following exposure to 0.036 µM thifluzamide; the expression of genes encoding ETC proteins, *ndufs4*, *sdha*, *uqcrc2*, *cox5ab*, and *atp5*α*1*, was significantly decreased in individuals exposed to 0.36 µM and above. Analysis of liver lipids revealed that thifluzamide decreased triglycerides (0.036 µM and above) and total cholesterol (0.36 µM and above). Also, fatty acid synthase (FAS) and carnitine palmitoyl transferase (CPT-1) activities were markedly decreased (0.36 µM). Importantly, hepatocyte damage was also observed (0.036 µM), including swollen endoplasmic reticulum (ER) and stripped ribosomes. Additionally, several genes related to lipid metabolism were markedly downregulated following exposure to thifluzamide at 0.036 (*prl*, *dgat1b*, *mgst1*, *insr*, and *ngf*) and 0.36 µM (*xdh*), while *ide* expression was significantly decreased in the two groups.

To help gain a better understanding of thifluzamide hepatotoxicity and the associated toxic mechanisms, Yang et al. [35] studied the effects of thifluzamide on the expression of genes related to hepatocyte physiology, unfolded protein response (UPR), and autophagy. The data showed that thifluzamide at 0.036 and 0.36 µM induced marked downregulation of hepatocyte-specific (*hpx*, *cyp3A4*, and *ces2*) and UPR genes (*atf6*, *ire1*α, *xbp1*, *perk*, *bip* and *atf4*), associated with increased expression of autophagy-related genes *lc3* (0.036 µM and above) and *beclin* and *agt-5* (0.36 µM). In addition, autophagic features in hepatocytes, but decreased caspase-3 activity, were observed following exposure to thifluzamide at 0.036 µM and above. These data suggest that oxidative stress and autophagy, along with ER stress, but not apoptosis, play an important role in the hepatotoxicity of thifluzamide in zebrafish.

To further analyze the adverse outcome pathways associated with the long-term toxicity of thifluzamide, Yang et al. [36] investigated the effects on growth and expression of genes encoding proteins involved in growth and development. They first found that the length and weight of adults were significantly decreased following exposure for 28 days to thifluzamide at 0.036 and 3.6 µM, respectively. The glucagon content was either decreased (0.036 and 0.36 µM) or increased (3.6 µM), growth hormone content was increased (0.36 µM), leptin accumulation was significantly decreased (0.036 µM and above), protein kinase A content was reduced (0.36 µM and above), and phosphorylated CREB levels were markedly increased (0.036 µM and above). Gene expression analysis also indicated marked changes in the expression of genes related to lipid metabolism and bone development in the liver. In particular, increased expression of *igf-1*, *lepa,* and *redd2*, combined with downregulation of *srebf*, *sirt1*, *nr1h3*, *apoa1*, *cav-1a*, *cav-1b*, and *redd1*, was observed in adults exposed to 0.036 and 0.36 µM. These findings demonstrate that thifluzamide caused severe leptin deficit associated with altered expression of genes related to growth and development.

Lastly, following the same 28-day exposure protocol, Yang et al. [37] re-analyzed cell apoptosis, mitochondrial damage, and expression changes of genes related to the hepatotoxicity of thifluzamide. The results first confirmed that thifluzamide at 0.036 µM induced an increase in the number of apoptotic and necrotic hepatocytes, combined with a decreased number of living cells and mitochondrial membrane potential. However, in contrast to previous findings [34], the results indicated marked upregulation of genes related to mitochondrion replication and transcription and ETC complexes: *polg1*, *twk*, *tk2*, *polmt*, *tfam*, *mt-nd1*, *ndufs4*, *uqcrc2*, *cox5ab*, and *atp5*α*1* (0.036 and 0.36 µM). Also, expression of the *sdha-d* genes was significantly decreased (0.36 and 3.6 µM), while the immunity-related *chitinase* (*chia*.) genes *chia. 1*, *chia. 4*, *chia. 5*, and *chia. 6,* and cell proliferation marker genes *cd3*, *cd45*, *ki67*, and *pak1* were markedly upregulated (0.036 and 0.36 µM). Importantly, a docking study showed that thifluzamide potentially binds to the SDH quinone binding site, but also to chitinase, albeit with lower affinity, making SDH a likely target of thifluzamide in zebrafish and chitinase a possible and unsuspected off-target of this SDHI.

## 6. Neurotoxicity and Behavior Deficits Induced by SDHI Fungicides

All animals, including fish, need a fully differentiated and functional central nervous system to find food, escape predators, reach adulthood and sexual maturity, and, ultimately, have offspring. Consequently, any neurotoxicant impairing neuron proliferation, axon pathfinding, synapse formation, axon myelination, neurotransmission, or any other process required for brain functioning can be detrimental to a species in the wild. However, it has long been known that the central nervous system is especially sensitive to toxic insults [38]. In particular, owing to the essential requirement for aerobic energy metabolism in the proper functioning of brain neurons, these cells constitute a likely target for pesticides whose mode of action relies on the inhibition of the mitochondrion ETC, such as SDHI fungicides. In addition, during brain development, a large number of finely regulated processes take place in the absence of a fully functional blood–brain barrier, making the developing brain particularly susceptible to neurotoxicants [38]. However, as highlighted below, few studies have so far investigated the neurotoxicity of SDHI fungicides and especially their adverse effects on neurodevelopment and behavior following low-dose long-term exposure.

The neurotoxicity of bixafen was first investigated by Li et al. [15]. They showed that the expression levels of the *neuroD* and *crx* and *sox2* genes linked to early neurogenesis were significantly downregulated after exposure to 0.3 and 0.9 µM bixafen, respectively, while *nkx2.4b* was upregulated (0.9 µM). In addition, downregulation of genes encoding proteins involved in cell cycle processes was observed in embryos exposed to 0.9 µM bixafen, suggesting that microcephaly of zebrafish embryos was at least partially caused by cell cycle inhibition. We also showed [16] that exposure to bixafen at 0.2 and 0.5 µM for 96 h induced dose-dependently reduced locomotion of embryos, likely the result of defective innervation of body muscles by motoneuron axons, which failed to properly innervate trunk muscles. The data confirmed that exposure to bixafen 0.2 and 0.5 µM also caused microcephaly.

The adverse effects of boscalid on CNS development and functioning were investigated in two recent studies. First, Wang et al. [39] showed that embryos exposed for 48 h to boscalid at 14.56 µM and above displayed gross brain defects, including decreased number of newborn neurons, enlarged brain ventricles, and reduced number of spontaneous movements. In addition, 6 dpf larvae exposed for 24 h to boscalid at 14.56 µM displayed markedly decreased locomotion. Using environmentally relevant concentrations, Qian et al. [31] found that larvae exposed for 7 days to boscalid at 0.87 and 1.74 µM showed significant inhibition of locomotor abilities and reduced phototactic response, respectively. Following 4 or 8 days of exposure to boscalid at 1.74 µM, larvae also showed decreased AChE activity and defects in cerebellar granule cell and retina neuron differentiation. Long-term toxicity studies (21 days) of boscalid toward adults indicated that exposure to 2.9 µM caused significant inhibition in average velocity and acceleration, but a significant increase in active time and distance moved, and exposure to 0.029 µM markedly impaired predatory abilities. Lastly, transcriptome analysis indicated changes in the expression of genes related to neurodevelopment in embryos exposed to bixafen at 1.74 µM for 96 h or 0.87 µM for 8 days, with downregulation of *mbp* and *synapsinIIa*, and upregulation of *gap43*. In addition, several genes required for eye development and phototransduction, *opn1sw1*, *opn1mw1*, *opn4.1*, and *rho,* were significantly upregulated following exposure to 0.87 µM boscalid for 8 days but downregulated with higher concentrations (3.49 µM). Exposure to subacute doses of boscalid thus impaired several essential neuro-behavioral processes, locomotion, and the ability to detect prey, possibly caused by visual system defects and a severe reduction in cerebellar granule cells.

The neurotoxicity of flutolanil toward embryos was investigated by Yang et al. [18]. The results first showed that genes involved in the circadian rhythm were significantly downregulated in embryos exposed for 96 h to flutolanil at 0.38 µM and above. The data also indicated that dopamine content was markedly increased (1.54 µM), the number of spontaneous movements was decreased (0.38 µM and above), and the expression of the *mao*, *th*, and *dbh* genes, encoding proteins involved in neurotransmitter synthesis, was significantly decreased (0.38 and 6.19 µM). Yang et al. [18] also showed that flutolanil at 0.38 µM markedly decreased the number of spontaneous movements of embryos and the expression of many genes encoding both positive and negative regulators of circadian rhythm: *clock1a*, *bmal1a*, *bmal1b*, *bmal2*, *aanat2*, *per1b*, *per2*, *per3*, *cry1aa*, *cry1ab*, *cry1ba*, and *cry1bb*.

The adverse effects of penthiopyrad on behavior were described by Qian et al. [21]. The data showed that embryos exposed to penthiopyrad at 0.83 µM for 8 days showed markedly reduced swimming velocity, acceleration speed, distance moved, and inactive time.

Yao et al. [30] investigated the adverse effects induced by sedaxane on embryos and observed microcephaly in individuals exposed to 6.35 µM and above for 5 days. However, further studies are needed to characterize the adverse outcome pathways involved.

Yang et al. [37] first observed that embryos exposed to thifluzamide 3.6 µM for 96 h displayed severe brain morphology defects. In particular, a marked reduction in the number of neurons was detected in the optic tectum and cerebellum. Also, Yang et al. [37] found that following 96 h exposure to thifluzamide at 3.6 µM, embryos displayed a dramatic decrease in dopamine content and major changes in the expression of genes involved in circadian rhythm, with increased expression of *clock1a*, *per1a*, *per1b*, *per2*, *per3*, *cry1aa*, *cry1ab*, *cry1ba*, *cry1bb, cry2*, and *cry3*. Thifluzamide at 0.36 µM also caused upregulation not only of *clock2*, *bmal1a*, *balm2*, *aanat2 per2*, *cry1ba*, and *cry1bb*, but also of *mao* and *dbh*, involved in neurotransmitter synthesis. These data show that thifluzamide, like flutolanil, may cause disruption of circadian rhythms, which are essential for proper fish behavior in wild environments.

## 7. Conclusions

This review confirms first that all SDHI fungicides for which the long-term toxicity has been extensively studied in zebrafish induce adverse effects, sometimes at environmentally relevant concentrations, such as bixafen (0.2 µM), boscalid (29 nM), isopyrazam (70 nM), flutolanil (0.77 and 150 nM), and thifluzamide (36 nM); this emphasizes the need to further and thoroughly investigate the consequences of long-term, low-dose exposure to the other studied SDHIs and to all the other 14 SDHI fungicides for which the toxicity to zebrafish has not been investigated so far. Importantly, while most data suggest that the toxicity of SDHI relies on the disruption of glycometabolism and energy production, leading to ROS production and, ultimately, to cell apoptosis, the identification of chitinase as a possible molecular target of thifluzamide highlights the existence of possible additional and unsuspected targets. Extensive and unbiased RNAseq investigations will be needed to characterize all the defects and deficits related to the toxicity of these SDHIs and to identify associated adverse outcome pathways.

## Figures and Tables

**Table 1:** LC_50_ values of SDHIs determined in zebrafish.

SDHIs	96 h LC_50_	Stages
**Bixafen**	2.12 µM 2.7 µM	embryo embryo
**Boscalid**	7.72 µM 4.85 µM	embryo adult
**Flutolanil**	16.91 µM 12.65 µM 12.09 µM 8.35 µM	embryo larvae (144 hpf) larvae (84 hpf) adult
**Fluxapyroxad**	1.83 µM 2.4 µM 3.64 µM	larvae adult embryo
**Isopyrazam**	0.14 µM	embryo
**Penthiopyrad**	7.70 µM 6.62 µM	embryo larvae
**Sedaxane**	11.7 µM	embryo
**Thifluzamide**	7.93 µM 6.66 µM 5.83 µM	adult larvae embryo

**Table 2:** LC_50_ values of SDHIs determined in fish species other than zebrafish.

SDHIs	96 h LC_50_	Species
**Benodanil**	19.8 µM	*Oncorhynchus mykiss*
**Benzovindiflupyr**	8.7 nM	*Cyprinus carpio*
**Bixafen**	0.23 µM	*Oncorhynchus mykiss*
**Boscalid**	7.86 µM	*Oncorhynchus mykiss*
**Fenfuram**	54.66 µM	*Poecilia reticulata*
**Fluindapyr**	0.34 µM	unknown species
**Fluopyram**	2.47 µM	*Coleonyx variegatus*
**Flutolanil**	16.7 µM	*Lepomis macrochirus*
**Fluxapyroxad**	0.76 µM 1.22 µM 3.02 µM 1.43 µM	*Cyprinus carpio* *Pimephales promelas* *Lepomis macrochiris* *Oncorhynchus mykiss*
**Furametpyr**	4.67 µM	*Cyprinus carpio*
**Isofetamid**	6.31 µM	*Oncorhynchus mykiss*
**Isoflucypram**	0.17 µM	*Oncorhynchus mykiss*
**Isopyrazam**	0.17 µM	*Cyprinus carpio*
**Mepronil**	37.13 µM	*Oncorhynchus mykiss*
**Oxycarboxin**	74.44 µM	*Oncorhynchus mykiss*
**Penflufen**	0.32 µM	*Cyprinus carpio*
**Penthiopyrad**	0.81 µM 1.07 µM 1.59 µM	*Pimephales promelas* *Oncorhynchus mykiss* *Cyprinus carpio*
**Pydiflumetofen**	0.42 µM	*Oncorhyncus mykiss*
**Sedaxane**	3.48 µM 1.96 µM	*Oncorhynchus mykiss* *Cyprinus carpio*
**Thifluzamide**	2.46 µM	*Lepomis macrochirus*
